# Pharmacological effects of novel microvesicles of basil, on blood glucose and the lipid profile: a preclinical study

**DOI:** 10.1038/s41598-021-01713-5

**Published:** 2021-11-11

**Authors:** Branislava Teofilovic, Svetlana Golocorbin-Kon, Nebojsa Stilinovic, Nevena Grujic-Letic, Aleksandar Raškovic, Armin Mooranian, Hani Al-Salami, Momir Mikov

**Affiliations:** 1 Department of Pharmacy, Faculty of Medicine, University of Novi Sad, Hajduk Veljkova 3, 21000 Novi Sad, Serbia; 2 Department of Pharmacology, Toxicology and Clinical Pharmacology, Faculty of Medicine, University of Novi Sad, Hajduk Veljkova 3, Novi Sad, Serbia; 3 Biotechnology and Drug Development Research Laboratory, Curtin Medical School, Curtin Health Innovation Research Institute, Curtin University, Perth, WA Australia

**Keywords:** Drug regulation, Pharmacology, Natural variation in plants

## Abstract

Microencapsulation represents a process that can create targeted, controlled release kinetics of drugs, thus optimizing therapeutic efficacy. Our group has investigated the impact of this technology on Wistar rats to determine pharmacological efficacy of basil extracts. Animals were treated with water extract of *Ocimum basilicum* in microvesicles and with combination of basil extracts and 3α,7α-dihydroxy-12-keto-5-cholanate, also known as 12-monoketocholic acid (MKC) acid in microvesicles for 7 days. Alloxan was used to induce hyperglycemia. Pharmacological effects on glycemia were evaluated by measuring blood glucose levels in alloxan-induced diabetic rats. Microvesicles were prepared using the Büchi-based microencapsulating system developed in our lab. The dose of basil extract that was orally administered in rats was 200 mg/kg and the dose of MKC acid was 4 mg/kg as per established protocols. A seven-day treatment with basil aqueous extract, as well as a combination of basil and MKC acid extract in the pharmaceutical formulation, led to a statistically significant reduction in the blood glucose concentration of animals with alloxan-induced hyperglycemia compared to pre-treatment values (p < 0.05 and p < 0.01), which indicates that basil has hypoglycemic and antihyperglycemic effects. Microvesicles, as a pharmaceutical-technological formulation, substantially enhance the hypolipidemic action of basil extract with MKC acid.

## Introduction

Microencapsulation can improve efficiency of drug loading and key manufacturing parameters, thus potentiating new avenues for pharmaceuticals as therapeutics in the healthcare markets worldwide. The new formulation systems created via microencapsulation technology control pharmacokinetics, pharmacodynamics, immunogenicity, nonspecific toxicity, and drug efficacy, and represent an interdisciplinary approach that combines polymer science, pharmaceuticals, bioconjugate chemistry, and molecular biology^1,2^. The main rationale behind novel drug delivery systems and encapsulation technologies is to avoid all the disadvantages of traditional drug transport in the body^3^. Many new carriers for drug delivery and targeting are developed to minimize drug degradation, drug loss, to prevent drug side effects, and to increase drug bioavailability^4,5^ Microencapsulation is a process that enables prolonged drug release and reduced side effects^4,6–8^.

When using herbal medicines, many components are destroyed due to the low pH in the stomach, while others can be metabolized in the liver before reaching the site of action. Consequently, the therapeutic effect will be absent if the compound of interest degrades preliminarily or is extensively metabolized previously. Natural components are metabolized much faster and easier in the body, so they cause fewer side effects compared to synthetic components^1^. As a result, the pharmaceutical industry is increasingly interested in herbal preparations. Herbal medications are often insoluble, so incorporating them into alginate microvesicles increases their bioavailability. Soluble plant materials can also be incorporated into microparticles to increase the bioavailability^9^.

The lack of appropriate drug formulations can represent the problem related to the control of the blood glucose levels^10^. Moreover, a knowledge of the pharmacokinetics and pharmacodynamics of antidiabetic medicines is crucial to enhance individualized drug therapy^11^. Modern therapy for diabetes mellitus lowers glycemic levels, but effectiveness fades after a certain period^10^. Therefore, the alternative ways to control glycemia and prevent complications caused by diabetes are the subject of the latest research related to the improvement of diabetes pharmacotherapy.

Numerous studies have shown that the effect of basil extract on blood glucose levels includes hypoglycemic and antihyperglycemic effects^12,13^. The hypoglycemic and antihyperglycemic effects of aqueous basil extracts can be attributed to the high content of total phenols and flavonoids. It is known that flavonoids, as strong antioxidants, can prevent progressive damage of pancreatic β-cells functions, caused by oxidative stress and thus reduce the occurrence of type II diabetes as native pancreatic β-cells lack intrinsic redox-defense mechanisms. They can also protect against the development of complications due to sustained hyperglycemia^12^. According to the literature, the antihyperglycemic action of basil extract is a consequence of inhibition of α-amylase and α-glucosidase activity, which slows glucose resorption from the intestinal tract and prevents postprandial hyperglycemia^12^, as well as effects on target tissue cells of insulin, on which basil potentiates the uptake of glucose from the blood, identical to the action of biguanides and thiazolidinediones^14^.

Bile acids, surfactants, chelating agents, and fatty acid derivatives are also known as substances that increase the trans-mucosal absorption of the drugs. Bile acid salts are well-known agents that enhance trans-membrane uptake of endogenous and exogenous lipids in the gastrointestinal tract, as well as trans-membrane and paracellular passage of small polar endogenous and exogenous molecules^8,15^. Numerous studies indicate that bile acids influence glucose metabolism and have hypoglycemic and antihyperglycemic effects. Bile acids, especially extensively studied 12-monoketocholic acid (MKC), inhibit the transcription of genes necessary for the synthesis of the enzyme phosphoenolpyruvate carboxykinase, which is crucial in the process of hepatic gluconeogenesis. This opens new areas of research on substances important for regulating glucose homeostasis in patients with diabetes^16^. Since basil preparations have their effect on glucoregulation primarily by increasing the utilization of glucose in peripheral tissues and do not burden the endocrine pancreas, it is necessary to determine their effectiveness in further studies as auxiliary medicinal products in patients with diabetes mellitus.

Formulation of the drug into smaller particles—micro dimension has a strong impact because it allows strictly controlled release of the drug substance and reduction of side effects, so the main goal of this study was to examine the effect of basil extract, traditionally prepared in alginate microcapsule formulation, on glucose regulation and lipid status in healthy and hyperglycemic experimental animals.

## Results and discussion

### Body weight values

After assigning the animals to control and experimental groups by random selection before the treatment, there was no statistically significant difference in body weight between groups of laboratory animals except between control groups treated with saline and basil extract and the control group treated with basil in the form of microvesicles (Table 1). This difference can be explained by the fact that the animals were taken at different times of the year, as well as that they were treated differently before coming to the Department of Pharmacology, Toxicology and Clinical Pharmacology. The approximately equal weight of animals before treatment favored the optimal performance of the experiment. The alginate micro carriers produced by the method of Mooranian et al. were used in this study^7^*.* At the end of treatment, the bodyweight of animals treated with aqueous basil extract in microvesicles after alloxan administration was significantly lower (210.6 ± 38.3 g) than the bodyweight of the control group of saline-treated animals (275.5 ± 30.7 g, p < 0.05). Moreover, the most significant decrease in the body weight was seen in the group of diabetic animals treated with basil extract in the microvesicles formulation (− 30.8 ± 8.0 g) in comparison with control group (p < 0.05). In animals treated with alloxan, alloxan insulin-dependent diabetes was induced. Since insulin is a strong anabolic hormone, in conditions of its deficiency, there is increased catabolism of proteins and fats^17^. Additionally, glucagon and adrenaline increase the catabolism of proteins and fats even more, so this can explain the weight loss observed between these groups. Apart from the group treated with alloxan and basil in the form of microvesicles, in all other groups, basil extract prevented statistically significant decrease in body weight. Furthermore, the protective effect of basil can be observed through the increase of body weight in control groups treated with MKC (+ 53.0 ± 8.2 g), combination of basil extract and MCK (+ 51.3 ± 5.2 g) and basil in microvesicles (+ 52.0 ± 4.6 g) in comparison with control groups treated with saline or basil alone (p < 0.05).

**Table 1:** Bodyweight values ((g), $\overline{\mathrm{x} }$ ± SD) in the control and experimental groups (6 animals per group).

	Group	Saline	Basil	MKC	Basil + MKC	Basil (micro)	Basil + MKC (micro)
Before treatment	Control	258.0 ± 20^a^	253.3 ± 16.6^a^	228.5 ± 28.9	229.3 ± 17,15	214 ± 13.8	233.3 ± 17.5
	Alloxan	263.3 ± 15.9	248.8 ± 20.1	232.5 ± 15.7	253.8 ± 15.4	241 ± 40.4	229.5 ± 34.0
After treatment	Control	287.3 ± 15.2	280.3 ± 13.6	281.5 ± 35.6	280.7 ± 19.6	266 ± 16.1	273.3 ± 24.8
	Alloxan	275.5 ± 30.7	253.5 ± 20.5	234.7 ± 29.1	257.7 ± 16.3	210.6 ± 38.3^b^	228.5 ± 53.7
Bodyweight change	Control	29.3 ± 13.7	27.0 ± 6.1	53.0 ± 8.2^**c**^	51.3 ± 5.2^**c**^	52.0 ± 4.6^**c**^	40.0 ± 14.8
	Alloxan	12.2 ± 18.2	4.7 ± 11.6	2.2 ± 28.4	3.8 ± 7.4	−30.8 ± 8.0^b^	−1.0 ± 33.7

^**a**^p < 0.05 in relation to the control group treated with basil (micro).

^**b**^p < 0.05 in relation to the alloxan group treated with saline.

^**c**^p < 0.05 in relation to the control group treated with saline, or basil.

### Blood glucose levels

**Figure 1:**
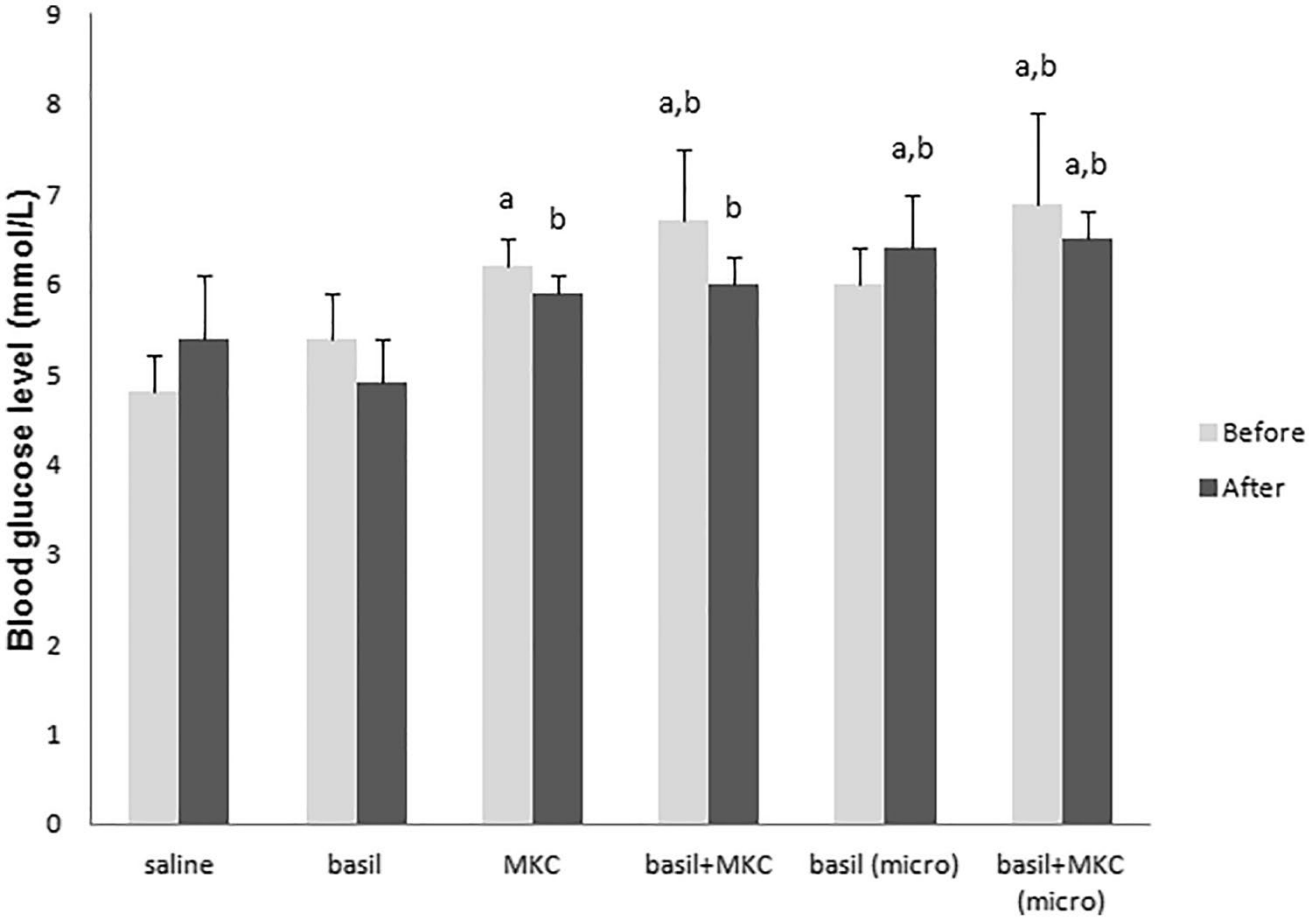
Blood glucose levels in normoglycemic animals before and after seven days of treatment; ^a^p < 0.05 in relation to the group treated with saline; ^b^p < 0.05 in relation to the group treated with basil.

**Figure 2:**
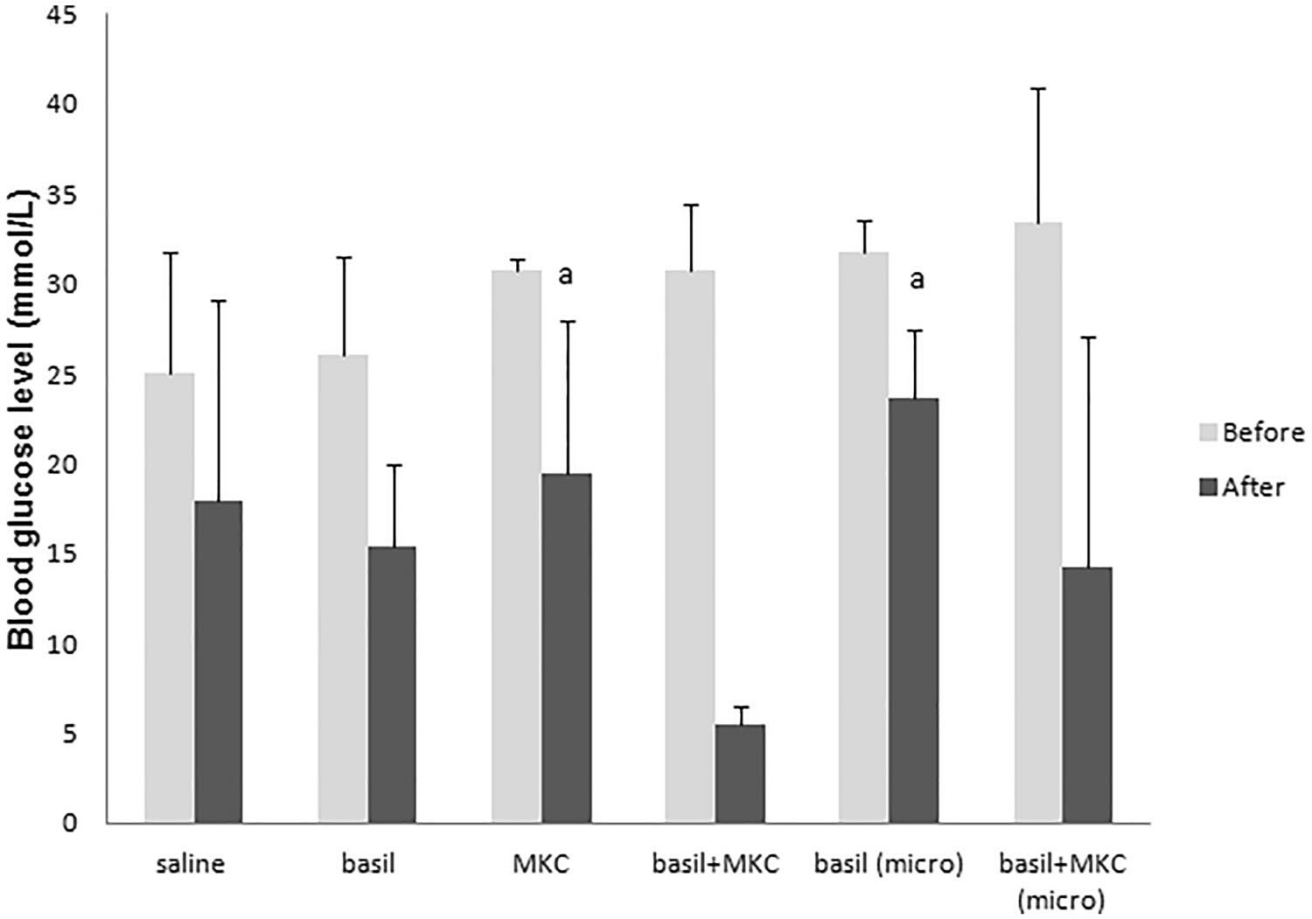
Blood glucose levels in diabetic animals before and after seven days of treatment; ^a^p < 0.05 in relation to the group treated with basil and MKC.

Figures 1 and 2 show the blood glucose levels in normoglycemic and diabetic animals (mmol/l, $\overline{x }$ ± SD), before and after seven days of treatment. Normoglycemic animals treated with a basil extract for seven days, had statistically lower values of glucose blood level at the end of the experiment compared to the animals treated with MKC alone, the combination of basil and MKC, basil in microvesicles and with combination of basil and MKC in microvesicles formulation (p < 0.05, Fig. 1), which is in agreement with similar study^18^. According to the results of other authors, aqueous basil extract can instantly reduce the glycemia of normoglycemic rats, but this does not leave long-term consequences on glycemia. The reason for this is preserved regulatory mechanisms of normoglycemic animals, primarily reduced glucose consumption and stimulation of gluconeogenesis in the liver^19^. However, basil extract in combination with bile acid caused an antihyperglycemic effect even in normoglycemic animals. In diabetic animals treated with the combination of basil and MKC the blood glucose level returned to normoglycemic values. This was statistically significant in comparison with the groups of animals treated with MKC alone and basil in microvesicles (p < 0.05, Fig. 2). End values of blood glucose are not so relevant in assessing antidiabetic effects as glycemic changes, and a special table of glycemia change was accordingly made to show the effect (Table 2). Treatment with basil and bile acids, alone or in combination, led to reduction of blood level even in normoglycemic animals. This change is more obvious in diabetic animals. Glucose blood level was lower at the end of treatment in all groups, and the difference was statistically significant in group treated with the combination of basil and MKC (− 25.2 ± 3.5) compared with group treated with saline (− 7.1 ± 8.3), basil alone (− 10.6 ± 7.0), MKC alone (− 11.2 ± 5.2) and with basil in microvesicle formulation (− 8.2 ± 4.1, p < 0.05). The obtained results coincide with the data of other studies in which basil exhibited hypoglycemic and antihyperglycemic effects^13,20,21^.

**Table 2:** Blood glucose level change in normoglycemic and diabetic animals after seven days of treatment.

Group	Saline	Basil	MKC	Basil + MKC	Basil (micro)	Basil + MKC (micro)
Normoglycemic	0.6 ± 1.0	− 0.5 ± 0.5	− 0.3 ± 0.5	− 0.7 ± 0.6^a^	0.4 ± 0.4	− 0.4 ± 1.1
Diabetic	− 7.1 ± 8.3^b^	− 10.6 ± 7.0^b^	− 11.2 ± 5.2^b^	− 25.2 ± 3.5	− 8.2 ± 4.1^b^	− 16.7 ± 11.7

^**a**^p < 0.05 in relation to the group treated with saline.

^**b**^p < 0.05 in relation to the group treated with basil + MKC.

### Lipid status

Table 3 presents the concentrations of triglycerides, total cholesterol, HDL and LDL cholesterol (mmol/l,$\overline{x }$± SD) and the serum index of atherosclerosis in normoglycemic animals and animals with alloxan-induced hyperglycemia, after seven days of *per os* treatment. The use of aqueous basil extract in normoglycemic animals did not lead to statistically significant changes in lipid status compared to the control group (treated with saline). However, the values of total cholesterol and LDL cholesterol were statistically higher after treatment with MKC alone (1.77 ± 0.29 mmol/l; 0.56 ± 0.12 mmol/l), combination of basil and MKC (1.88 ± 0.20 mmol/l; 0.56 ± 0.11 mmol/l) and basil extract applied in the form of microvesicles (1.63 ± 0.37 mmol/l; 0.43 ± 0.15 mmol/l) in comparison with groups treated with saline (1.17 ± 0.20 mmol/l; 0.20 ± 0.08 mmol/l) and basil extract alone (1.04 ± 0.30 mmol/l; 0.20 ± 0.10 mmol/l). The concentration of HDL cholesterol was significantly greater in groups of normoglycemic animals treated with MKC alone (0.94 ± 0.15 mmol/l), combination with basil and MKC (0.99 ± 0.08 mmol/l) and basil in microvesicles formulation (0.89 ± 0.21 mmol/l) compared to animals treated with basil alone (0.59 ± 0.19 mmol/l). This indicates the protective effect of basil extract and bile acid salts, as well as their synergistic action. The use of a combination of basil extract and MKC in the form of microvesicles led to a statistically significant decrease in triglyceride concentration (0.31 ± 0.17 mmol/l) compared to normoglycemic animals treated with saline (0.57 ± 0.14 mmol/l), MKC alone (0.64 ± 0.14 mmol/l), combination of basil and MKC (0.66 ± 0.10 mmol/l) and with basil extract in microvesicles formulation (0.60 ± 0.17 mmol/l), (p < 0.05). After treatment with basil extract alone, triglyceride concentration was also decreased compared to the other groups, but without statistical significance. The atherosclerosis index was statistically significantly higher in the groups treated with MKC (0.58 ± 0.07 mmol/l), the combination of basil extract and MKC (0.58 ± 0.11 mmol/l), and the combination of basil extract and MKC in the form of microvesicles (0.57 ± 0.14 mmol/l), compared to the control group (0.28 ± 0.07 mmol/l) and the group treated with basil extract (0.35 ± 0.05 mmol/l), p < 0.05. Lower concentration of triglyceride, total and LDL cholesterol were achieved after treatment with basil extract, compared to all other groups, indicates the hypolipidemic effect of basil even in normoglycemic animals.

**Table 3:** The levels of triglycerides, total cholesterol, HDL and LDL cholesterol (mmol/l, $\overline{\mathrm{x} }$ ± SD) and serum index of atherosclerosis in normoglycemic and diabetic animals.

	Group	Saline	Basil	MKC	Basil + MKC	Basil (micro)	Basil + MKC (micro)
Normoglycemic animals	TGC	0.57 ± 0.14^a^	0.50 ± 0.09	0.64 ± 0.14^a^	0.66 ± 0.10^a^	0.60 ± 0.17^a^	0.31 ± 0.17
	Total chol	1.17 ± 0.20	1.04 ± 0.30	1.77 ± 0.29^a,b,c^	1.88 ± 0.20^a,b,c^	1.63 ± 0.37^b,c^	1.25 ± 0.14
	HDL	0.70 ± 0.14	0.59 ± 0.19	0.94 ± 0.15^c^	0.99 ± 0.08^a,b,c^	0.89 ± 0.21^c^	0.70 ± 0.09
	LDL	0.20 ± 0.08	0.20 ± 0.10	0.56 ± 0.12^b,c^	0.56 ± 0.11^b,c^	0.43 ± 0.15^b,c^	0.40 ± 0.11
	Aterogenic index	0.28 ± 0.07	0.35 ± 0.05	0.58 ± 0.07^b,c^	0.58 ± 0.11^b,c^	0.48 ± 0.07^b^	0.57 ± 0.14^b,c^
Diabetic animals	TGC	0.89 ± 0.99	0.55 ± 0.17	1.06 ± 0.35	0.54 ± 0.10	0.48 ± 0.42	0.55 ± 0.34
	Total chol	0.96 ± 0.20	1.07 ± 0.55	2.01 ± 0.30^b,c^	2.20 ± 0.29^b,c^	1.56 ± 0.44	1.65 ± 0.65
	HDL	0.56 ± 0.15	0.58 ± 0.31	1.11 ± 0.15^b,c^	1.18 ± 0.18^b,c^	0.95 ± 0.26	0.94 ± 0.37
	LDL	0.34 ± 0.21	0.25 ± 0.23	0.41 ± 0.27	0.75 ± 0.13^b,c^	0.41 ± 0.21	0.48 ± 0.13
	Aterogenic index	0.45 ± 0.32	0.36 ± 0.16	0.36 ± 0.19	0.65 ± 0.05	0.42 ± 0.18	0.50 ± 0.11

^**a**^p < 0.05 in relation to basil + MKC (micro).

^**b**^p < 0.05 in relation to the group saline.

^**c**^p < 0.05 in relation to the group basil.

The concentration of total cholesterol was statistically significantly higher in diabetic animals treated with MKC (2.01 ± 0.30 mmol/l) and basil extract in combination with MKC (2.20 ± 0.29 mmol/l), both concerning the control group (0.96 ± 0.20 mmol/l) and the group treated only with basil extract (1.07 ± 0.55 mmol/l), p < 0.05. The concentration of HDL cholesterol was statistically significantly higher in diabetic animals treated with monoketocholic acid (1.11 ± 0.15 mmol/l) and basil extract in combination with MKC (1.18 ± 0.18 mmol/l), compared to the control group (0.56 ± 0.15 mmol/l) and the group treated with basil extract alone (0.58 ± 0.31 mmol/l), p < 0.05. The lowest value of LDL cholesterol in diabetic animals was measured in the group treated with aqueous basil extract. In the group treated with a combination of MKC and basil extract (0.75 ± 0.13 mmol/l), the concentration of LDL cholesterol was statistically significantly higher compared to the control group (0.34 ± 0.21 mmol/l), as well as in the group treated with basil extract alone (0.25 ± 0.23), p < 0.05. There is no statistically significant difference in the atherosclerosis index between the control group and experimental groups of animals with alloxan-induced hyperglycemia. In this study, individual treatment with aqueous basil extract alone (0.36 ± 0.16 mmol/l), and in a microvesicule formulation (0.42 ± 0.18 mmol/l), and MKC (0.36 ± 0.19 mmol/l), reduced the atherosclerosis index in a group of animals with alloxan-induced hyperglycemia, but the difference was not statistically significant. Treatment with basil extract alone and basil extract in combination with monoketocholic acid in diabetic animals caused a decrease in triglyceride concentration (0.55 ± 0.17 mmol/l; 0.54 ± 0.10 mmol/l) and increase values of the HDL cholesterol (0.58 ± 0.31 mmol/l; 1.18 ± 0.18 mmol/l). The use of MKC alone or in combination with basil increased the values of total cholesterol, HDL and LDL. Aqueous basil extract and basil extract in combination with MKC in the microvesicles formulation lowered the triglyceride value compared to the control group and increased the HDL cholesterol value, but the difference was not statistically significant. The beneficial effect of aqueous basil extract on the lipid status of diabetic animals is explained by the fact that phenolic components and flavonoids of basil potentiate cholesterol clearance by inducing up-regulation of LDL receptors, and using basil extract, inhibit the activity of hydroxymethyl-glutaryl-CoA reductase, enzymes on whose activity the synthesis of endogenous cholesterol largely depends^22,23^. Although both the literature and our study show clear results of the beneficial effect of basil extract and bile acids, especially in the form of microvesicles on glucose metabolism, as well as the results of basil extract on the lipid status of animals with alloxan-induced diabetes, the results of MKC indicate the need for additional studies of both endogenous and synthetic bile acid derivatives that would fully elucidate their effect on lipid metabolism.

## Experimental

### Chemicals

Alloxan (CAS number 2244–11-3, molecular weight 160.08) and alginate (CAS number 9005–38-3, molecular weight 216.12) were obtained from Sigma-Aldrich (USA). Calcium chloride and all other chemical reagents and solvents were purchased from Merck (Germany).

### Plant material and preparations

The commercial sample of aerial parts of *Ocimum basilicum* L. 1753 was purchased from the Institute for Medicinal Plant Research "*Dr Josif Pancic*", Belgrade, Serbia). Voucher specimen was confirmed and deposited at the Herbarium BUNS of the University of Novi Sad, at the Department of Biology and Ecology, Faculty of Sciences. (no. 2-1518). The air-dried plant material was milled in blender and mean particle size was determined by sieve set (CISA Cedaceria Industrial, Spain) to be 0.3 mm. Extract was prepared as infusion by mixing 1 g of dry plant material with 200 ml of boiling water, with occasional stirring, and it was extracted for 10 min (according to recommendations and instructions). The final step involved the entire contents being filtered through the filter paper (MN 616 md, 110 mm, Macherey–Nagel, Germany) and evaporated to dryness under vacuum. This extraction method was chosen because it provides extracts with the best phenolic profiles and antioxidant activities. Selected extract represents the way of preparing tea in everyday household. One of the strongest benefits of this extraction’s method is using water as a green extraction medium. It is inexpensive, non-hazardous, easily available, environmental-friendly solvent^24,25^.

### Pharmaceutical-technological formulation of the extract into microvesicles

Solution of dry basil extract of 200 mg/ml and MKC (4 mg/ml) were made in HPLC grade water. A solution of calcium chloride dihydrate (20 mg/mL) was made by adding anhydrous calcium chloride in HPLC pure water. The solution of sodium-alginate (30 mg/ml) was made in HPLC pure water. The stock solutions were mixed separately in a magnetic stirrer at room temperature for about 4 h and then stored in the refrigerator at + 4 °C to + 8 °C until further use. They were used within 48 h of making. Sodium salt of monoketocholic acid (MKC) (3α, 7α-dihdroxy-l2-oxo-5β-cholanate sodium salt) was prepared according to procedures by Kuhajda et al.^26^. Fresh solutions for the treatment of rats of basil extracts (200 mg/mL) and MKC (4 mg/ml) were prepared by dissolving the powder in ultrasonic suspending gel (10%), mixing for 2 h at 25 °C prior to administration to the animals. Alloxan powder (130 mg) were put in Eppendorf vials and reconstituted with normal saline solution (1 mL) immediately before the injection into the tail vein of rats, to induce Type 1 diabetes. Solutions of basil extract and MKC were used within 12 h of preparation and were stored in the refrigerator when not in use, as per protocols^27,28^.

Microvesicles of sodium alginate with determined substance were prepared using microencapsulating system designed in-house via BUCHI technologies (BÜCHI Labortechnik, Switzerland)^29–31^. Parameters were set in a frequency range of 1,000 to 1,500 Hz and constant flow rate of 4 ml/min. The alginate microvesicules were produced in our laboratory by the same technology as described in paper Mooranian et al.^7^. Polymer solutions containing sodium alginate and basil extract with or without MKC were made up to a final concentration (basil extract-MKC-sodium alginate) in a ratio of 1:3:30 respectively. This was based on our previously published paper. Two formulations were prepared, one with basil extract (200 mg/mL) in sodium alginate solution (30 mg/mL) and the other with basil extract (200 mg/mL) and MKC (4 mg/mL). Three series were prepared, combined, and used in animal experiments. All 3 series of microvesicles were prepared and treated in the same way. The microencapsulation efficiency was calculated as the percentage of encapsulated extract divided by the amount of total basil extract added^32^^,^^7^. The amount of basil extract loaded in alginate microvesicules was quantified by measuring of total phenolic by the method which is described in our previous paper^33^. The dose of basil extract administered orally to rats in the form of microvesicles was 200 mg/kg and the dose of MKC was 4 mg/kg. All solvents and reagents for making microvesicles were used without further purification.

### Laboratory animals and experimental procedures

72 male Wistar rats with bodyweight of 250–300 g, were used and selected by the method of random selection from the offspring of the Military Technical Institute Belgrade. Animal care and all experimental procedures have been performed in accordance with the EU Directive 2010/63/EU on animal welfare and under the Law on Animal Welfare of the Republic of Serbia (OG RS 41/09) and ARRIVE guidelines. All experimental procedures were carried out according to a protocol approved by the Ethics Commission for the protection of the welfare of laboratory animals of the University of Novi Sad (Novi Sad, Serbia; No. 01-90/4-4) and approval of Ministry of Agriculture and Environmental Protection (Belgrade, Serbia; No. 323-07-00550/2014-05 of January 28, 2014). All methods were performed in accordance with the relevant National and International guidelines and regulations. During the experiment, the animals were kept at the standard conditions for the laboratory animals in the Department of Pharmacology, Toxicology and Clinical Pharmacology of the Medical Faculty in Novi Sad. Room temperature was set to 20–25 °C, humidity 55% ± 1.5% with light and dark cycles that lasted 12 h each. The animals were given free access to food and water. A total of 72 animals were split randomly into 12 groups, each group consisting of 6 animals. Six groups were healthy, without alloxan pretreatment, and six groups were with alloxan-induced diabetes (AID). Healthy and diabetic rats were split into the following subgroups:

- control animals, treated with 0.9% saline solution, 1 ml/kg bw, p.o., for 7 days.
- experimental animals, treated with basil extract, 200 mg/kg bw, p.o., for 7 days.
- experimental animals, treated with MKC, 4 mg/kg, p.o., for 7 days.
- experimental animals, treated with the combination of basil extract (4 mg/kg bw) and MKC (4 mg/kg bw), p.o., for 7 days.
- experimental animals, treated with basil extract, 200 mg/kg bw, in microvesicles formulation, p.o., for 7 days.
- experimental animals, treated with the combination of basil extract (4 mg/kg bw) and MKC (4 mg/kg bw), in microvesicles formulation, p.o., for 7 days.

On the last day of experiments, 2 h after administration of the last dose of plant extracts the rats were anestethised with a 25% solution of urethane (Sigma Chemicals Co, St Louis, MO, USA), in a dose of 0.75 g/kg intraperitonealy (i.p.). After losing of the righting reflex, the animals were euthanized by cardiopuncture obtaining samples of blood and other tissues for further examination.

### Antidiabetic and biochemical activity

A solution of alloxan was used to induce hyperglycemia (Diabetes mellitus type 1) in experimental animals, or in other words alloxan diabetes, which is similar to insulin-dependent diabetes in humans to some extent. Still, this type of diabetes has some features of type II, as well, thanks to alloxan’s reactive oxygen species mechanism of beta cell toxicity which can be found in both diabetes types^34^. Thus, in order to destroy beta cells selectively alloxan powder was put in Eppendorf vials and reconstituted with 0.9% isotonic saline solution immediately before intraperitoneal administration in a single dose of 130 mg/kg. 48 h after the application of alloxan, the blood sample was taken from the tail vein and the concentration of glucose in the blood was measured. Animals with blood glucose concentrations greater than 15 mmol/l were included in the further course of the study. The concentration of glucose in the capillary blood sample, taken from the tail vein of rats, was determined by Accu-check Active device (Roche Basel, Switzerland). The blood glucose concentrations were measured immediately before the start of treatment, 48 h after the administration of alloxan (to determine whether diabetes was induced) as well as at the end of the experiment. After the treatment with saline, basil extract and/or MKC, and subsequent sacrifice of the animals, biochemical tests of lipid status were performed.

### Statistical analysis

Statistical processing of the in vivo obtained test results was performed by the statistical program IBM SPSS Statistics, version 21. The arithmetic mean ($\overline{x }$*)* was used as a measure of the central tendency of a group, and the measure of the variation among the data was expressed by the standard deviation (SD). One-way analysis of variance (ANOVA) was employed for the comparisons between experimental groups. Post- hoc testing for ANOVA was performed using Tukey’s HSD test. The difference between groups was considered statistically significant for a p-value less than 0.05 (p < 0.05).

## Conclusion

Microvesicles, as a pharmaceutical-technological formulation, significantly potentiate the hypolipidemic action of basil extract and MKC. The combination of fixed doses of basil extract and sodium salt of monoketocholic acid, applied in the form of microvesicles, showed the most notable decrease in the concentration of triglycerides in the serum of both normoglycemic and diabetic animals. Used in the form of microvesicles, basil extract statistically significantly increased the concentration of HDL cholesterol in the serum of diabetic animals. Since MKC itself has produced hypoglycemic and hypolipidemic effects, this synthetic derivative of bile acids is a substance whose use prevents disorders present in the metabolic syndrome. Therefore, the results of this study are the basis for future clinical trials to determine the therapeutic potential of MKC, in the form of the new pharmaceutical formulation.

## Data Availability

The datasets generated during and/or analysed during the current study are available from the corresponding author on reasonable request.
